# Point-of-care testing with CRP in primary care: a registry-based observational study from Norway

**DOI:** 10.1186/s12875-015-0385-8

**Published:** 2015-11-19

**Authors:** Ingrid K. Rebnord, Steinar Hunskaar, Sturla Gjesdal, Øystein Hetlevik

**Affiliations:** National Centre for Emergency Primary Health Care, Uni Research Health, Kalfarveien 31, 5018 Bergen, Norway; Department of Global Public Health and Primary Care, University of Bergen, Post box 7804, 5020 Bergen, Norway

**Keywords:** Point-of-care CRP testing, Primary care, GPs working-style, Children

## Abstract

**Background:**

Norwegian primary health care is maintained on the regular general practitioners (RGPs), GP’s contracted to the municipalities in a list patient system, working at daytime and at out-of-hours services (OOH services). Respiratory disease is most prevalent during OOH services, and in more than 50 % of the consultations, a CRP test is performed. Children in particular have a high consultation rate, and the CRP test is frequently conducted, but the contributing factors behind its frequent use are not known. This study compares the RGPs rate of CRP use at daytime and OOH in consultations with children and how this rate is influenced by characteristics of the RGPs.

**Methods:**

A cross-sectional register study was conducted based on all (*N* = 2 552 600) electronic compensation claims from consultations with children ≤ 5 year during the period 2009–2011 from primary health care. Consultation rates and CRP use were estimated and analysed using descriptive methods. Being among the 20 % of RGPs with the highest rate of CRP use at daytime or OOH was an outcome measure in regression analyses using RGP-, and RGP list characteristics as explanatory variables.

**Results:**

One third of all RGPs work regularly in OOH services, and they use CRP 1.42 times more frequently in consultations with children in OOH services than in daytime services even when the distribution of diagnosis according to ICPC-2 chapters is similar. Not being approved specialist, have a large number at their patient-lists but relatively few children on their list and a large number of consultations with children were significantly associated with frequent use of CRP in daytime services. The predictors for frequent CRP use in OOH services were being a young doctor, having many consultations with children during OOH and a frequent use of CRP in daytime services.

**Conclusions:**

The increase in the frequency of CRP test use from daytime to OOH occurs in general for RGPs and for all most used diagnoses. The RGPs who use the CRP test most frequently in their daytime practice have the highest rate of CRP in OOH services.

## Background

Primary health care in Norway is based on regular general practitioners (RGPs) with daytime practice contracted to the municipalities in a list patient system and are also supposed to take care of acute medical problems at daytime. The municipalities are also responsible for organizing an out-of-hours service (OOH service) which can be contacted by all inhabitants 24/7 when RGPs are not available, in afternoons, nights and holidays. When accessibility to RGPs is low, the use of OOH services increase [1]. The OOH organization varies from a single doctor on call in smaller municipalities to larger units serving more municipalities or the larger cities, with doctors and other healthcare professionals working together in casualty clinics [2]. The RGPs are obliged to take part in the OOH service and have approximately 50 per cent of all out-of-hours contacts; the rest are covered by physicians temporarily working in primary care as locums or residents or by hospital doctors. Only a small proportion (24 %) of the OOH doctors have finished a 5-year training program and are approved specialists in general practice [3, 4]. It has previously been shown that the RGPs working in OOH services have shorter consultations and request less laboratory analyses than do other doctors [5].

Norway has a high rate of contacts to the OOH services because of the gate-keeper function for secondary care, in contrast to other countries, where the patients can choose between emergency departments and OOH services [6–9]. The majority of contacts are related to infections and respiratory sickness (75 %), especially in the youngest age group and during the winter months [10]. Data recorded at 7 different OOH services in 2007 estimating national figures on use-pattern showed that 76 % of all contacts were considered as not urgent in a strict medical sense [11]. Small children (0–5 years) have an especially high contact rate (430/1000 inhabitants per year) [12] and infectious diseases dominate both the use of OOH services and the RGPs in daytime practice [13]. This patient group may therefore be appropriate to study how the use of CRP varies among RGPs in daytime practice and OOH services.

On-site measurement of C-reactive protein (CRP) is the most frequently used laboratory test in Norwegian OOH services [12]. The test aims at differentiating bacterial infections from viral/less severe infections, and after its introduction as a point-of-care test, it has been widely used in Norway: at 35 % of all consultations at OOH-services [10]. Compared to most other countries the CRP test is used clearly more frequent in Norway and reasons for such variations should be assessed, especially since the benefit of using the test has been discussed [14–16]. Different factors seem to influence use of the test: the age, gender and experience of the doctor, as well as the geographical centrality and organization of the OOH service [12]. The wide differences in use indicate that factors other than sickness or symptoms in the population may contribute to the variation. Economic incentive may be a factor, since the doctor is paid extra for conducting a CRP test. For OOH services, the municipalities most often cover the costs while the doctors keep the income. During daytime service, the RGPs most often cover the costs of services and retain the income from their own surgery practice, and thus the economic incentive per test is relatively low.

It is not known if the usage pattern of laboratory tests at OOH reflects the individual RGP’s overall working style or if it represents a change associated with working for the OOH services. When the RGPs work with their own patients at daytime, they have a different age mix, different list length, and are located in either rural or urban districts, which may affect the patients’ encounters with their RGPs [17]. In the OOH services, the RGPs meet a random group of patients. Difference in use from daytime to OOH can be explained with different prevalence of serious illness and different ways of organizing services, but there should in theory be no difference in the use of CRP between RGPs at OOH services if the use is based strictly on medical indications.

The aim of this study is therefore threefold: (1) to assess the use of CRP tests in consultations with children 0–5 years, (2) to compare the use of CRP in regular daytime practice and OOH services, and (3) to study associations between variations in the use of CRP and characteristics of the RGPs.

## Methods

The study is a cross-sectional, register-based, nationwide study in primary care in Norway. The material comprises all claims from consultations with children aged 0–5 years from RGPs’ daytime practice and from OOH services in 2009, 2010 and 2011.

Both the daytime RGP practices and OOH services are mainly financed by a fee-for-service system. The RGPs send a claim to the Norwegian Health Economics Administration (HELFO) for each patient contact, with information about the RGP’s identity, type of contact, daytime or OOH and eventually fees for laboratory tests or procedures. The claim also includes information about age and gender of the patient and a diagnosis based on ICPC-2 [18–20]. The term diagnoses in the Norwegian ICPC-2 are used for both symptoms/complaints and diseases like infections or injuries.

The fee for a consultation increases by approximately one third (NOK 92, i.e. approximately 12 Euros) when taking a CRP test, compared to a consultation without a laboratory test.

The HELFO data have been linked with information from the national RGP database that includes information about the individual RGP’s age, gender, speciality, list size, whether the list is open for new patients, and practice municipality.

The total material of 2 552 600 contacts with children aged 0–5 years formed the basis for describing the use of CRP in consultations. When comparing the RPGs’ practice in daytime and OOH, we included only the group of RGPs that had more than a total of 20 consultations by children 0–5 years during the three-year period of daytime service, and that also worked OOH during the same years and had more than a total of 20 OOH consultations with children (*N* = 1931). The RGP database has no information about doctors working as locums and residents in daytime practice or OOH services, therefore not included in all analyses.

### Ethical approvals

HELFO and The Norwegian Data Protection Authority allowed the use and linkage of data. The Norwegian Directorate of Health, as register owner, also approves the linkage of registers.

### Statistics

The data were analysed in IBM SPSS 21.0 using descriptive analyses, T-tests and regression analyses. To illustrate the distribution of mean CRP rates per RGP at daytime and OOH we used quintiles and cross tabulation. Being in the fifth quintile with the highest rate of CRP use daytime and OOH, respectively, was used as an outcome variable in the multivariable logistic regression models. Goodness-of-fit of the model was assessed by a Hosmer and Lemeshow test for different cut-off-values of the dependent variable but showed no differences if we used fourth and fifth quintile together or just fifth quintile, so the fifth quintile was chosen. The *p*-value in the test was 0.417 for a high CRP use at daytime and 0.474 for a high CRP use at OOH, assessing good fitness of the model chosen.

Explanatory variables in the multivariable logistic regression analyse were age, gender, specialist status of the RGP, total number of contacts daytime and OOH, CRP used per contact at daytime, size of patient list, whether the list was open and number of children in the list. They are chosen to test different theories of possible association using available relevant data in register and the full model is presented.

## Results

Table 1 shows the number of consultations and use of CRP by RGPs and other doctors in daytime and OOH services. CRP was used in 31 % of all consultations at daytime and in 44 % of all at OOH, and to a higher extent by doctors that were not RGPs (53 % at OOH). When selecting the 20 most used diagnoses, we found that respiratory diseases, infections and fever constituted 50 % and 59 % of all contacts at daytime and OOH, respectively, and a CRP test was used in 44 % and 58 % of the consultations. These 20 diagnoses represented 81 % of all CRP tests and the mean CRP rates for the RGPs with these diagnoses are shown in Table 2, all rates significant higher OOH compared to daytime.

**Table 1:** Distribution of all consultations in the regular general practice scheme with children 0–5 years at daytime and at out-of-hour services, and rate of CRP use during 2009–2011

	Total	RGPs also working OOH	RGPs not working OOH	Other doctors^a^
Consultations				
Daytime (n)	2 080 743	758 709	977 235	344 799
Daytime, distribution (%)	100	36	47	17
OOH (n)	471 857	251 246	0	220 611
OOH, distribution (%)	100	53	0	47
Rate of CRP use in Consultations				
Daytime	0.31	0.30	0.29	0.33
OOH	0.44	0.43	0	0.46

^a^Other doctors are locums, residents etc

**Table 2:** Distribution of mean CRP rate per diagnose for the regular general practitioners, the 20 most used diagnoses

Diagnoses	CRP rate daytime (SD)^a^	CRP rate OOH (SD)^a^	*P*-value
Fever	0.76 (0.20)	0.82 (0.22)	<0.001
Respiratory infection	0.70 (0.23)	0.82 (0.22)	<0.001
Pneumonia	0.75 (0.26)	0.88 (0.22)	<0.001
Influenza	0.74 (0.26)	0.86 (0.22)	<0.001
Bronchitis/bronchiolitis	0.69 (0.25)	0.82 (0.24)	<0.001
Acute upper respiratory infection	0.60 (0.23)	0.72 (0.26)	<0.001
Acute tonsillitis	0.70 (0.28)	0.78 (0.28)	<0.001
Viral infection	0.72 (0.25)	0.82 (0.25)	<0.001
Throat symptoms	0.69 (0.26)	0.84 (0.24)	<0.001
Streptococcal infection	0.70 (0.28)	0.78 (0.28)	<0.001
Cough	0.52 (0.24)	0.74 (0.27)	<0.001
Acute laryngitis	0.64 (0.29)	0.69 (0.29)	0.001
Gastroenteritis	0.60 (0.28)	0.74 (0.28)	<0.001
Vomiting	0.62 (0.28)	0.78 (0.25)	<0.001
Diarrhoea	0.49 (0.27)	0.81 (0.26)	<0.001
Conjunctivitis	0.14 (0.14)	0.28 (0.26)	<0.001
Otitis media	0.39 (0.25)	0.59 (0.31)	<0.001
Abdominal pain	0.41 (0.25)	0.78 (0.25)	<0.001
Asthma	0.41 (0.25)	0.78 (0.25)	<0.001
Urinary infection	0.79 (0.29)	0.94 (0.11)	<0.001

^a^ *SD* Standard deviation

Table 3 compares the RGPs working both in daytime practice and OOH services with RGPs working in daytime practice only. The RGPs working both places were younger, fewer were approved specialists in general practice, they were more often males and had fewer patients at their list. However, the use of CRP was not significant different.

**Table 3:** The study sample of regular general practitioners (RGPs) working also in out-of-hours (OOH) services^a^ compared with RPGs not working OOH in 2009–2011 (T-independent sample test)

Variable	RGP working OOH	RPG not working OOH	*P*-value
Number of RGPs	1931	2834	
RGP mean age, years	43	52	<0.001
Proportion male RGP (%)	67	64	0.005
Proportion approved specialist in general practice (%)	50	62	<0.001
Mean list size	1119	1231	<0.001
Mean number of consultations with children 0–5 years in the study period	391	345	<0.001
CRP rate per consultation at daytime (Standard deviation)	0.30 (0.13)	0.29 (0.14)	0.151

^a^Inclusion criteria: RGPs having >20 consultations with children 0–5 years OOH in the period

**Fig. 1:**
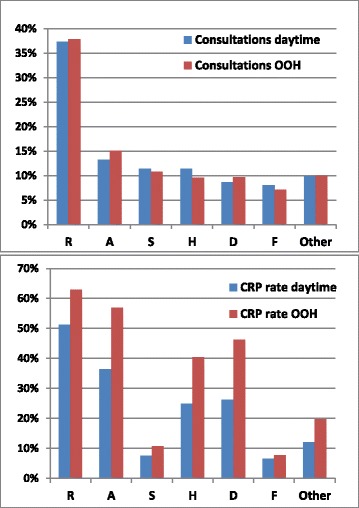
Distribution of all consultations and the frequency of CRP use per consultation with children 0-5 years in 2009-2011. The distribution is shown in percentage according to ICPC-2 chapters at the regular GP services at daytime (*n* = 2 080 743) and at out-of-hour services (*n* = 471 857). R: Respiratory, A: General and unspecified, S: Skin, H: Ear, D: Digestive, F: Eye, Other: All other diagnostic groups

The distribution of diagnoses at ICPC chapter level at daytime and OOH was rather similar (Fig. 1). The CRP rate was significant higher at OOH than at daytime in the total material, mean difference from daytime to OOH 0.14 (CI 0.09-0.19, *p* < 0.001) (not tabled).

### RGPs’ use of CRP

Table 4 shows the variation in the rate of CRP usage per RGP, distributed in quintiles. The accuracy for being in the same group in daytime and OOH services, if we accept a variance of one quintile, is 91.9 %. If the diagonal is considered as a strict constraint, 42.1 % of the doctors are in the same quintile for both daytime and OOH. The proportions over and under the diagonal are almost identical (28.8 % vs 29.0 %). Only a small minority (4.3 %) of the doctors with the highest rate of CRP at the OOH services had a low rate of CRP at daytime. Similarly, we found that only 2.9 % of the RGPs were both high users at daytime and in the lowest quintile at OOH. The 207 doctors (10.7 %) in the fifth quintile both at daytime and OOH used 23.0 % of all CRP tests at OOH and 18.0 % of all tests at daytime.

**Table 4:** Number of regular general practitioners (RGPs)^a^ in the study sample (*n* = 1931) distributed in quintiles by their mean use of CRP per out-of-hours (OOH) and daytime consultations

CRP rates at daytime (quintiles and rate intervals)		CRP rates at OOH (quintiles and rate intervals)					
		1	2	3	4	5	All
		<0.25 (0.18)	0.25 – 0.36 (0.31)	0.36–0.45 (0.41)	0.45–0.54 (0.50)	>0.54 (0.62)	
1	< 0.18 (0.13)	234	92	43	24	9	402
2	0.18 – 0.25 (0.22)	97	133	81	48	25	384
3	0.25 – 0.32 (0.29)	39	88	122	91	42	382
4	0.32 – 0.41 (0.37)	13	50	99	117	102	381
5	> 0.41 (0.47)	9	21	40	105	207	382
All		392	384	385	385	385	1931

Frequency rates are shown as mean number of CRP tests per consultation. Median value in ()

^a^RGPs having >20 consultations with children 0–5 years OOH in the period

### Predictors for high usage of CRP tests

A multiple regression analysis was performed in order to identify associations between characteristics of RGPs and a high CRP rate. We analysed predictors for being in the highest quintile of CRP use at daytime and OOH independently (Table 5). A high rate of CRP at daytime was associated with not approved RGPs, female RGPs, a larger list size, fewer children at list and a large number of consultations with children. We found that a high frequency rate of CRP at daytime was strongly associated with the same tendency at OOH. In addition, being a young doctor and having a large number of consultations with children were factors that were significantly associated with a high rate of CRP in OOH services.

**Table 5:** Associations between frequent use^1^ of CRP in daytime and out-of-hours (OOH) consultations with children (0–5 years) and characteristics of the regular general practitioners (RGPs), lists and practice (*n* = 1931 RGPs)

	Predictors at daytime			Predictors at OOH		
Variables	OR^a^	95 % CI^b^	P	OR^a^	95 % CI^b^	P
RGPs age (per year)	1.008	0.990–1.026	0.398	0.957	0.937–0.978	0.000
Female RGP^c^	1.437	1.023–2.018	0.037	1.134	0.776–1.657	0.516
Specialist in general practice^d^	0.700	0.507–0.967	0.031	0.915	0.641–1.307	0.626
Number of consultations at daytime, children 0–5 years (per 10 contact)	1.012	1.004–1.019	0.002	0.990	0.981–0.999	0.028
Number of consultations at OOH, children 0–5 years (per 10 contact)	1.002	0.990–1.014	0.723	1.026	1.013–1.040	0.000
List size (per 100)	1.111	1.054–1.170	0.000	1.016	0.958–1.079	0.592
Number of children 0–5 years on patient list (per child)	0.987	0.982–0.993	0.000	1.001	0.996–1.007	0.599
Closed patient list (yes/no)^e^	0.841	0.627–1.128	0.248	1.010	0.723–1.410	0.954
OOH consultations/daytime consultations (%)	1.001	0.997–1.005	0.682	0.998	0.994–1.003	0.492
CRP rate daytime (%)				1.119	1.101–1.137	0.000

1) Frequent use defined as being among the RGP with a CRP rate in the highest quintile in daytime practice and OOH respectively, see Table 3

^a^OR: Odds Ratio

^b^CI: confidence interval

^c^Male RGP is reference

^d^Not approved specialist is reference

^e^Open list is reference

Continuous variables: Age, contacts, number on patient list, rates in percent

## Discussion

### Main findings

This study from Norwegian primary care shows that 82 % of all consultations with children 0–5 years are at daytime and 18 % at OOH. Infectious diseases constitute 50 % of consultations in daytime practice and 59 % in OOH practice. CRP is used in 31 % of all consultations at daytime and in 44 % in OOH. RGPs not approved as specialists in general practice, female RGPs and larger list size are associated with more frequent use of CRP in daytime practice. The rate of RGPs’ use of CRP in daytime practice seems to be an important predictor for the use of CRP in OOH services.

### Strengths and limitations

This material is comprehensive and is based on three successive years; all electronic claims from RGPs and OOH services are included. The paper based claims that are not included are estimated to be 2 per cent in 2009 and less than 1 per cent from 2010 [10], selection bias is therefore minimal, and the results can be seen as representative for Norwegian general practice.

Children under 6 years of age are a homogenous group of patients with a high contact rate with both RGPs and OOH services. The distribution of diagnoses was similar at daytime and OOH, according to the diagnose chapters in ICPC-2. Children seldom have chronic diseases, but some planned controls for asthma and other diseases may explain some more consultations at daytime not taking a CRP. Still we find the reasons for contact at daytime and OOH are comparable, thereby enabling a comparison of RGPs work daytime and OOH.

A limitation is that the validity of diagnoses used in RGP claims is not known. The RGPs may give a more severe diagnosis when the CRP is high. Because of this verification bias the diagnoses should not be used as an explanation for variation in CRP.

Another limitation is that there are rather few consultations and CRP tests included for some RGPs who work less frequently in OOH services. The great difference between the number of consultations at daytime and OOH results in a less reliable basis for comparison. We also have no information about the doctors working as locums in the registry; however as a group they work a lot in OOH services and use more CRP.

Because doctors are paid extra for performing CRP tests, that may be an incentive for taking CRP. The difference between daytime practice and OOH services is that the doctors are not responsible for the actual cost of the CRP test at most OOH services. However, this may vary and our data has no information about which RGPs must cover the cost for the CRP test kit, and this lack of information can be considered a limitation.

In 2009, the contact rate for respiratory infections was especially high, probably due to the swine influenza pandemic during that year, but the increase was equally distributed between the RGPs at daytime and OOH [21].

### Comparison with existing literature

#### Use of CRP

During the past decade, there has been increased awareness regarding the problem of antibiotic resistance and the high level of prescriptions in primary care for self-limiting infections. Diagnostic uncertainty is a major problem, and the CRP used as a point-of-care test has shown reduced antibiotic prescription in some studies [22–27]. Studies from Sweden have shown that CRP was used in 36–42 % of respiratory infections in 2005 [15, 28] and that is the same level we found at daytime services in our study. However, at OOH we found the usage rate of CRP to be almost 60 % for respiratory infections. There are no studies that make a conclusion of cut-off values for CRP level and when antibiotics are recommended [15, 16].

#### RGP’s experience

Two thirds of all RGPs had no contacts with OOH or so few that they cannot be considered to have regular duties in OOH services [29]. To be a young RGP was correlated with a high use of CRP in OOH, and to not be an approved specialist was correlated with high use in daytime service. We think that this reflects the fact that experience is an important factor in the diagnostic process; older doctors are more often specialists, and they use CRP to a lesser extent. A patient list including a larger number of children was associated with a lower rate of CRP and may also be explained by the RGPs having a greater degree of experience with paediatric problems.

#### Economy

A high total number of patients and a high rate of daytime contacts with children indicate a doctor working a lot, having longer days and/or more days with patient contact per week. Financial motivation may be relevant, but another explanation may be that an effective working method is to perform the laboratory test as a routine before the consultation and thereby avoid having to wait for the lab results after the consultation. Among RGPs who work regularly in OOH services, there seems to be a small group of doctors working a lot; these are younger RGPs, but their lists of patients do not exceed a mean of 1200 patients. It probably reflects a group of young doctors with a high working capacity and use OOH-services to increase their income [30]. Having many consultations OOH was also significantly associated with frequent use of CRP and may indicate that the financial motivation matter.

### Implication for practice

The use of CRP especially in OOH services is high and may reflect an acquired practice to routinely perform a CRP test when the patient has fever or an infection. Studies have shown that CRP may have an effect at reducing prescription when a lower respiratory infection is suspected [22] and since the diagnoses are given at the end of the consultation when the result is ready and the decision of treatment is taken, this may reflect the high level of CRP for diagnoses as fever, cough, respiratory infections, pneumonia, influenza, bronchiolitis and upper respiratory infections. For other diagnoses as sore throat, tonsillitis and otitis the test are known to be of little value [31], still the use in Norway is high. There exist no guidelines in Norway for when the CRP-test is indicated. The guidelines for antibiotic treatments in primary care [32] give some advice for what level of CRP to suspect bacterial infections in lower respiratory infections but for throat symptoms the guidelines recommend a strep A test, so according to this there seem to be an overconsumption of CRP.

There seems to be different factors that can explain the increase in CRP use in Norway. We have mentioned the financial motivation and the doctor’s experience, but it must also be taken into account that the organization of the services may play a role. During recent years, OOH services are increasingly organized in larger districts, with many patients treated at short time and only one or very few doctors, assisted by ancillary staff who routinely may take the tests before the consultation. There is a risk involved in placing one’s trust in the test alone, for both parents and health personnel, when used to this degree.

An earlier study has shown that RGPs do not change practice style when moving to a new patient population [33]. The strong association between the RGPs’ use of CRP in daytime and OOH indicates that they use CRP to an extent that is more a kind of working style for many doctors rather than as a test that is medically indicated.

Our study indicate that to increase the awareness concerning the medical indications for taking laboratory tests is recommended to prevent excessive use. Removal of the financial incentive may reduce the use, but more studies are needed to find more correct medical indications for taking CRP in children and are important for preventing overconsumption.

### Further research

This study does not give any information on the usefulness of the CRP in selecting the best treatment for patients or reducing the use of antibiotics. In further studies, focus should be on clinical findings and treatment, to ascertain whether the use of a CRP test results in less or more use of antibiotics. The clinical significance of CRP in primary care needs to be further investigated.

## Conclusions

The point-of-care test CRP is frequently used all over in primary care and all doctors use it more in the OOH-services than in daytime practice. The RGPs that most frequently use CRP tests in daytime service do the same in OOH services. Being a young doctor and having a high number of consultations result in significantly higher use of CRP in OOH services. The differences between the RGPs use of CRP in OOH services cannot be explained by different diagnoses.

## Abbreviations

CRP: C reactive protein
GP: General practitioner
RGP: Regular general practitioner
OOH: Out-of-hours
